# The Heating and Lighting of Hospital Wards

**Published:** 1909-02-13

**Authors:** 


### HOSPITAL ADMINISTRATION.

### CONSTRUCTION AND ECONOMICS.

### THE HEATING AND LIGHTING OF HOSPITAL WARDS.

#### IV.—THE RELATIVE ADVANTAGES AND COST OF THE DIFFERENT METHODS OF HEATING.

Of all the methods of heating available, the Plenum, considered solely as a method of heating, is by far the most efficient. The Plenum system, if properly carried out, provides an atmosphere at any temperature the doctor desires, in any ward. Further, if properly carried out, it causes no draughts, and no changes of temperature in going from wards to corridors, day rooms, etc., and *vice versâ*; and it carries off all offensive effluvia, such as are present at dinner and other times. For the operating theatre also it is the only system that will maintain the required temperature in all parts of the theatre and adjoining rooms without any attention from those in the room. The least expensive method of heating is by means of hot water or steam radiators, and if the heat for the hot water or steam can be provided by exhaust steam, which has already done work in an engine, the heating should be carried out very economically, though the heat is not so well distributed as with the Plenum system. Next to the hot-water or steam-heated radiators, warming by means of the air-heating stoves is the most economical, and there is a great deal to be said for a combination of these stoves, placed at the ends and the middle of the ward, with steam or hot-water radiators placed round the ward. The open coal fireplace is the most expensive but one, and by far the least efficient, in the sense that it does the least to provide a uniformly warmed atmosphere. The most expensive method of heating at the present price of electricity is by means of electric radiators.

The relative costs run in the following ascending order: steam and hot-water heated radiators, heated-air stoves, the Plenum system, open fireplaces, and electric radiators. The cost of heating by gas is about the same as by the air-heating stoves. But this hardly tells the whole tale. As in every case where human agency is employed, there is a vast difference between examples of the various systems named. The Plenum system may be made comparatively inexpensive, or may be made comparatively expensive, according to the arrangement of the plant. It is, in any case, hardly adapted for small hospitals, convalescent homes, etc., mainly because of the heavy cost of building the ducts that are required; and this particularly applies to buildings already in existence. For large new hospitals it is practicable to arrange ducts so that the air current is efficient, and yet does not absorb too much power. Mr. Henman, the architect of the Birmingham General and Belfast Victoria Hospitals, who has successfully applied the Plenum system in those hospitals, states that in planning a hospital to use the Plenum system it is possible to save money on the ground covered. The Plenum system suffers from the necessity for building large ducts, and also from the somewhat complicated apparatus, requiring a skilled attendant. This, in the writer's opinion, puts it out of court for small hospitals. Further, it requires the expenditure of power to drive the air through the ducts, and if the ducts are improperly designed, if they are small in comparison with the volume of air they are to carry the power required may be considerable. Further, if the ducts are small, it is sometimes difficult to avoid unpleasant draughts, because the velocity of the air through them is necessarily great, and it is not always possible to reduce it to comfortable limits in the rooms to be warmed. For small hospitals, or convalescent homes, probably the most economical, the most convenient, and that requiring least attendance, if properly put in, is the hot-water system, the water being heated in a boiler in the basement, designed for heating water only, not for converting water into steam. There are many such boilers on the market, which require very little attention, being almost self-feeding, and the working costs of heating and of supplying of water for the ordinary requirements of patients, nurses, etc., is as low as it can be brought. Hot-water radiators are better than steam radiators for general use, because the temperature of the surface of the former is lower than that of the steam-heated radiator. With high temperatures there is the tendency to an unequal distribution of heat, and also to the burning of any dust which may be present in the atmosphere, may settle upon the radiator, and produce a most unpleasant odour. The early steam radiators also were somewhat difficult to regulate. Modern apparatus, however, has overcome those difficulties, and with proper arrangement and proper attendance it is almost a matter of the individual convenience and individual taste, whether steam or hot water should be employed.

The electric radiator is put completely out of court for heating wards by reason of its enormous expense. At the lowest price at which electricity is sold for heating purposes, or at which it can be generated by the hospital's own plant, the cost of heating would be very many times greater than the most expensive of the other systems. Perhaps the following will give some idea of the reason of this. One heat unit will raise the temperature of 55 cubic feet of air 1° F. One lb. of coal in burning liberates from 10,000 heat units upwards. One cubic foot of ordinary illuminating gas in burning gives out 600 heat units. One Board of Trade electrical unit, the kilowat, as it is called, gives out only 57 heat units. At the lowest price the Board of Trade electrical unit costs 1d.; 1 cubic foot of gas at 2s. 6d. per 1,000 cubic feet costs $\frac{3}{100}$d.; and 1 lb. of coal, at 25s. per ton, costs a little over $\frac{1}{10}$d. The heat delivered by the electric radiator is all utilised, while that delivered by gas and coal is only partially utilised, but the difference, when everything in favour of electricity has been taken into account, is still enormous. On the other hand in small rooms where heating is only required for a short time, electric radiators are convenient and inexpensive, because the actual cost of the current used is small. Gas-heated flueless radiators can also be used for the same purpose, and are very economical even for comparatively large rooms.

